# Aqueous core epigallocatechin gallate PLGA nanocapsules: characterization, antibacterial activity against uropathogens, and *in vivo* reno-protective effect in cisplatin induced nephrotoxicity

**DOI:** 10.1080/10717544.2022.2083725

**Published:** 2022-06-16

**Authors:** Badriyah Alotaibi, Thanaa A. El-Masry, Engy Elekhnawy, Aya H. El-Kadem, Asmaa Saleh, Walaa A. Negm, Dalia H. Abdelkader

**Affiliations:** a Department of Pharmaceutical Sciences, College of Pharmacy, Princess Nourah Bint Abdulrahman University, Riyadh, Saudi Arabia; b Pharmacology and Toxicology Department, Faculty of Pharmacy, Tanta University, Tanta, Egypt; c Pharmaceutical Microbiology Department, Faculty of Pharmacy, Tanta University, Tanta, Egypt; d Department of Biochemistry, Faculty of Pharmacy, Al Azhar University, Cairo, Egypt; e Pharmacognosy Department, Faculty of Pharmacy, Tanta University, Tanta, Egypt; f Pharmaceutical Technology Department, Faculty of Pharmacy, Tanta University, Tanta, Egypt

**Keywords:** Double emulsion/solvent evaporation, sustained-release, dual surfactant action, cisplatin, NLPR-3 inflammasome, Nrf-2, KIM-1

## Abstract

Epigallocatechin-3-gallate (EGCG) was isolated from *Cycas thouarsii* leaves for the first time and encapsulated in aqueous core poly(lactide-co-glycolide) (PLGA) nanocapsules (NCs). This work investigates antimicrobial activity and *in vivo* reno-protective effects of EGCG-PLGA NCs in cisplatin-induced nephrotoxicity. A double emulsion solvent evaporation process was adopted to prepare PLGA NCs loaded with EGCG. Particle size, polydispersity index (PDI), zeta potential, percent entrapment efficiency (%EE), structural morphology, and *in vitro* release platform were all studied *in vitro*. The optimum formula (F2) with particle size (61.37 ± 5.90 nm), PDI (0.125 ± 0.027), zeta potential (–11.83 ± 3.22 mV), %EE (85.79 ± 5.89%w/w), initial burst (36.85 ± 4.79), and percent cumulative release (87.79 ± 9.84) was selected for further *in vitro/in vivo* studies. F2 exhibited an enhanced antimicrobial activity against uropathogens as it had lower minimum inhibitory concentration (MIC) values and a more significant impact on bacterial growth than free EGCG. Forty male adult mice were randomly allocated into five groups: control vehicle, untreated methotrexate, MTX groups treated with a daily oral dose of free EGCG, placebo PLGA NCs, and EGCG PLGA NCs (F2) for 10 days. Results showed that EGCG PLGA NCs (F2) exerted promising renoprotective effects compared to free EGCG. EGCG PLGA NCs group induced a significant decrease in kidney index, serum creatinine, kidney injury molecule-1 (KIM-1), NGAL serum levels, and pronounced inhibition of NLPR-3/caspase-1/IL/1β inflammasome pathway. It also significantly ameliorated oxidative stress and decreased NFκB, Bax expression levels. Aqueous core PLGA NCs are a promising formulation strategy that provides high polymeric protection and sustained release pattern for hydrophilic therapeutic agents.

## Introduction

Epigallocatechin-3-gallate (EGCG) is a polyphenolic molecule isolated from different plants (Zhang et al., 2020; Alam et al., 2022; Buchmann et al., 2022). Several studies have shown that EGCG has anti-inflammatory, anti-oxidative, and anti-fibrotic properties, implying that it may be a preventative tool in different metabolic illnesses and its consequences. It was found that EGCG could enhance organ protection against injury (Sulistiyani et al., 2021). Basically, renal tissues have a high possibility for acute injury due to several etiologies. One could be attributed to bacterial infection leading to urinary tract infections (UTIs). The main uropathogens causing UTIs are *Escherichia coli*, *Klebsiella pneumoniae*, *Pseudomonas aeruginosa*, and *Proteus mirabilis* (Flores-Mireles et al., 2015). UTI could lead to sepsis or septic shock. In addition, it could cause sudden deterioration of renal function and acute kidney injury. Despite many antibiotics, the spreading resistance among bacterial isolates imposes the high need for a continuous search to develop novel antimicrobial drugs.

Additionally, severe renal dysfunction and acute kidney tissue injury are also associated with administering multiple chemotherapeutic agents such as cisplatin. Cisplatin is a common anti-neoplastic medication used to treat malignancies such as breast, ovarian, lung, head and neck, and urinary bladder. Although most chemotherapy regimens include it, nephrotoxicity is a prevalent side effect limiting its efficacy (Mehmood et al., 2014). As a result, minimizing kidney injury in cisplatin-treated patients is critical, and the pathogenesis of cisplatin should be elucidated to design a new medicine to moderate the nephrotoxicity induced by cisplatin (Aldossary, 2019). Despite current research into the pathophysiological foundation of cisplatin nephrotoxicity, the molecular mechanism of cisplatin-induced kidney injury has yet to be determined. However, necrosis, oxidative stress, inflammation, and apoptosis may play essential roles in cisplatin-induced nephrotoxicity (Pabla & Dong, 2008).

Although EGCG has various pleiotropic features, its high hydrophilic properties negatively affect its effectiveness (Andreu Fernández et al., 2020). Generally, health-enhancing aspects of EGCG are reduced after oral intake due to its poor intestinal stability and permeability (Granja et al., 2019). Furthermore, EGCG has an outstanding liability for chemical degradation at higher pH of the small intestine, leading to structural instability (Andreu Fernández et al., 2020). Several works of literature demonstrate that absolute oral bioavailability of EGCG could be extremely low, reaching 0.1% after oral administration (Pandit et al., 2019; Li et al., 2020). Recently, there has been an urgent need to create a novel nano vesicular system that could entrap EGCG, promoting its permeability and stability (Li et al., 2020).

Formulation of nanocapsules (NCs) could be adjusted depending on the nature of their centric core, which might be aqueous or organic, allowing lipophilic or hydrophilic substances to be entrapped. Several types of a biodegradable and biocompatible polymeric material such as poly(d,l-lactide) PLA, poly(d,l-glycolide) PLG, and their copolymers poly(lactide-co-glycolide) (PLGA) are widely utilized to formulate nano- and microparticles (Faheem & Abdelkader, 2020). These polymers have been approved for clinical use with high safety levels. Recently, one of the formulation's challenges was the design and formulation of aqueous-core NCs encapsulating a high proportion of water-soluble therapeutic agents. To date, the w/o/w multiple emulsion method, which is based on two strategies, nanoprecipitation or emulsion-diffusion methods, has been employed for this purpose. This technique necessitates using a stabilizing agent such as polyvinylpyrrolidone (PVP), polyvinyl alcohol (PVA), tween, or a combination between more than one surfactant to ensure perfect system homogeneity (Cosco et al., 2015; Dimer et al., 2020).

Herein, for the first time, we isolate the EGCG from *Cycas thouarsii* R.Br. The development and physicochemical characterization of novel aqueous-core PLGA NCs are discussed. PLGA NCs were prepared to utilize PLGA with a molecular weight of 24–38 kDa and a combination of hydrophilic and lipophilic nonionic surfactants. EGCG was used as a candidate for a hydrophilic drug. Its entrapment efficiency, *in vitro* release, and reno-protective effects on cisplatin-induced nephrotoxicity were evaluated and compared with the free drug in albino mice as an animal model. In addition, we evaluated the antimicrobial activity of EGCG-PLGA NCs and free EGCG against different uropathogens.

## Materials and methods

### Plant materials, extraction, and isolation of EGCG

*Cycas thouarsii* R.Br. leaves (Cycadaceae) were obtained from El-Abd Garden in Giza in Jan 2017. Dr Esraa Ammar, Plant Ecology Department, Faculty of Science, Tanta University, Tanta, Egypt, kindly confirmed the plant identification. A voucher specimen (PGG-W-004) was preserved at the Pharmacognosy Department. The powdered leaves (1750 g) were extracted with methyl alcohol (four times × 5 L). The extract was concentrated using a rotary evaporator to obtain a total extract residue. The total methanolic extract (70 g) was suspended in CH_3_OH:H_2_O (50%), then partitioned with *n*-hexane, CH_2_Cl_2_, ethyl acetate, and then *n*-butanol saturated with H_2_O yielding different fraction residues, respectively (Negm et al., 2021).

Ethyl acetate fraction (2.8 g) was subjected to a silica gel column chromatography (*ϕ* 2.5 × 70 cm, 100 g silica, collected fraction 30 mL) using gradient elution, starting with CH_2_Cl_2_, and increasing polarity using CH_3_OH to obtain five fractions (E1:E5). Fr. E1 (CH_2_Cl_2_:CH_3_OH; 96:4 eluate), Fr. E2 (CH_2_Cl_2_:CH_3_OH; 94:6 eluate), Fr. E3 (CH_2_Cl_2_:CH_3_OH; 92:8 eluate), Fr. E4 (CH_2_Cl_2_:CH_3_OH; 90:10 eluate), and Fr. E5 (CH_2_Cl_2_:CH_3_OH; 86:14 eluate). Fr. E5 (935 mg) was isocratically chromatographed using silica gel, eluted with 90% CHCl_3_. Sub-fractions (22–31, 310 mg) were collected and purified on Sephadex LH-20 eluted with 100% CH_3_OH to get a white amorphous powder of compound **I**.

### Chemicals and reagents

Poly (d,l-lactide-co-glycolide, acid terminated, lactide:glycolide 50:50), PLGA, MW 24–38 kDa, and PVA, poly(vinyl alcohol) (MW = 31–50 kDa, 87–89% hydrolyzed) were obtained from Sigma-Aldrich (Gillingham, UK). Oxoid™ phosphate buffered saline tablets were purchased from Thermo Fisher Scientific (Waltham, MA). Tween 80 was supplied from El Nasr Pharmaceutical Chemicals Co. (Cairo, Egypt). Span 80 was obtained from Oxford Lab Fine Chem LLP (Navghar, India). Oleic acid was manufactured and packed by Piochem for Laboratory Chemicals (October City, Egypt). Dimethylformamide (DMF), sodium lauryl sulfate (SLS), MeOH, CH_2_Cl_2_, ethyl acetate, and *n*-butanol were purchased from Al-Gomhoria Company (Cairo, Egypt). We employed silica gel F254 (Merck, Kenilworth, NJ, 70–230 mesh), and Sephadex LH-20 (Sigma-Aldrich Chemical Co., St. Louis, MO) for column chromatography. Cisplatin injection was obtained from Mylan Pharmaceuticals Co. (Bengaluru, India).

### Spectral techniques

A JEOL ECA500-II-NMR spectrometer (Akishima, Japan) recorded NMR spectra at 500 MHz for ^1^H and 125 MHz for ^13^C. DMSO-d_6_ was utilized to dissolve the NMR sample. The chemical shifts were normalized using solvent resonances. Thermo Scientific's ISQ Quantum Access MAX Triple Quadrupole system (Waltham, MA), Xcalibur 2.1 software, and USA Mass Spectrometer were used for the ESI-MS.

### Preparation of polymeric nanocapsules with an aqueous core

PLGA NCs with aqueous core were fabricated using modified double emulsion/solvent evaporation method (Abdelkader et al., 2018; Almukainzi et al., 2022). An ultrasonic probe sonication (Cole-Parmer Model 50 Cp T 4710 Series, Vernon Hills, IL) was adjusted at 40% of its highest power in an ice bath, EGCG (10 mg) (Zhang et al., 2020) aqueous solution was added to DMF organic phase (2 mL) containing PLGA, oleic acid, and span 80 (Cosco et al., 2015) (Table 1), then sonicated for 2 min. The primary emulsion was resonicated in an aqueous (5 mL) solution containing Tween 80 (Cosco et al., 2021) and PVA (Table 1). The nanodispersion was agitated for three hours to evaporate DMF, and the obtained EGCG-PLGA NCs were washed three times with distilled water using Hettich Microliter Centrifuge MIKRO 220 (Kirchlengern, Germany) at –4 °C and 15 × 10^3^ rpm for 20 min per cycle. The pellets of PLGA NCs were then collected for further *in vitro* experiments.

**Table 1.** Key formulae, composition, and formulation variables of EGCG-PLGA NCs.

Formula key	Internal aqueous core	Organic interface			External aqueous shell		Internal: organic: external phases’ volume ratio
		Span 80 (%w/v)	PLGA (%w/v)	Oleic acid (%w/v)	Tween 80 (%w/v)	PVA (%w/v)	
F1	EGCG (10 mg) dissolved in distilled water	1	0.75	20	1	0.5	0.04:0.4:1
F2			1.5				
F3			3				

### Physical characterization of EGCG-PLGA NCs

The effects of PLGA concentration, at three levels (Table 1), on the physical characterizations of EGCG-PLGA NCs in terms of particle diameter, size distribution, surface charge, percent entrapment efficiency (%EE), and *in vitro* release pattern were thoroughly investigated.

#### Surface charge, particle size, and polydispersity index (PDI)

The homogeneity, size distribution, presence of residue or precipitates, and phase separation of EGCG-PLGA NCs dispersions were all examined. The average diameter and PDI were assessed using dynamic light scattering (Zetasizer Nano, NanoBrook 90Plus, Brookhaven Instruments Corporation, Holtsville, NY). The electrophoretic mobility was used to determine the zeta potential utilizing folded capillary cells using the same instrument. All measurements were carried out in triplicate at room temperature (25 °C). The data are expressed as mean ± SD.

#### Entrapment efficiency (EE % w/w)

EGCG entrapped in the aqueous core of PLGA NCs was indirectly measured by analyzing the amount of free EGCG that escaped to the outer shell of PLGA NCs. Using UV–vis spectrophotometry (Evolution 300 spectrophotometer, Thermo Scientific, Basingstoke, UK) at 275 nm (detection wavelength) (Huang et al., 2019), the amount of EGCG that retained in the supernatant during the centrifugation step of PLGA NCs fabrication was quantified as follows (Almukainzi et al., 2022):

$$\text{EE}(\%w/\text{w) }=\frac{\text{total}\text{mass}\text{of}\text{drug}\text{used}-\text{mass}\text{of}\text{drug}\text{in}\text{the}\text{supernatant}}{\text{total}\text{mass}\text{of}\text{drug}\text{used}}\times 100$$

### Transmission electron microscopy

The structural morphology of EGCG-PLGA NCs was investigated using a transmission electron microscope (TEM, JEM-2100 Electron Microscope, JEOL Ltd., Akishima, Japan). Before visualization, PLGA NCs samples were mounted with copper grids covered with carbon film and stayed till drying. All measurements were accurately displayed using Image J program (Bethesda, MD).

### *In vitro* release of EGCG from aqueous core PLGA NCs

EGCG release was investigated using cellulose acetate dialysis tubing (molecular weight cut off 12–14 kDa, Fisher Scientific, Waltham, MA), tightly secured with clips as previously mentioned (Abdelkader et al., 2021; Almukainzi et al., 2022). EGCG-PLGA NCs (1 mL) were packed in a dialysis bag, which was subsequently placed into a beaker containing 200 mL of the release buffer mainly composed from SLS (1%, w/v) (Almukainzi et al., 2022) dissolved in PBS (pH = 7.4) (Cosco et al., 2015), allowing sink conditions to be maintained for 24 hours during the experiments. At various time intervals, 2 mL samples were removed and replaced with an equal volume of new release media. Then, the samples were subjected to UV–visible spectrophotometry, as described before. There was no interference between the absorbance peaks of EGCG and the various components that formulate PLGA NCs. Therefore, the release of free EGCG was assessed to examine the influence of PLGA NCs on the sustained release pattern. All *in vitro* release studies were carried out in triplicate, and the percent cumulative release values were presented as + SD.

### *In vitro* antibacterial activity

#### Bacteria

There are four standard bacterial strains: *Escherichia coli* ATCC 25922, *Klebsiella pneumoniae* ATCC 13883, *Pseudomonas aeruginosa* ATCC 27853, and *Proteus mirabilis* ATCC 35659 were utilized for investigation of the antibacterial activity of the free EGCG, EGCG PLGA NCs (F2), and placebo PLGA NCs. These bacterial strains were obtained from Pharmaceutical Microbiology Department, Pharmacy Faculty, Tanta University, Tanta, Egypt.

#### Susceptibility to free EGCG, EGCG PLGA NCs, and placebo PLGA NCs

The antibacterial activity of free EGCG, EGCG PLGA NCs (F2), and placebo PLGA NCs was elucidated using cup plate method (CLSI, 2014). Briefly, 100 µL of each bacterial suspension was spread on the surface of Mueller–Hilton agar plates. Then, five wells were done by a sterile cork-borer and 100 μL of each: free EGCG, EGCG PLGA NCs (F2), and placebo PLGA NCs, 10% dimethyl sulfoxide (negative control), and ciprofloxacin (positive control) were added in each well. The concentrations of free EGCG and EGCG encapsulated into PLGA NCs (F2) were kept to be equivalent (1000 μg/mL).

#### Detection of the values of minimum inhibitory concentration (MIC) of free EGCG and EGCG PLGA NCs

We determined the MIC values for free EGCG and EGCG PLGA NCs (F2) against the tested isolates using the broth microdilution method (Attallah et al., 2021). We determined the MIC values by detecting the lowest concentration, which resulted in the complete absence of growth. In addition, microtitration plates had wells that contained bacterial suspension only (positive control) and well-contained broth only (negative control).

#### Impact on the bacterial growth process

The impact of free EGCG and EGCG PLGA NCs (F2) on the growth of the tested bacterial isolates was inspected using 0.5 MIC values (Attallah et al., 2021; Elekhnawy et al., 2021). We detected the values of the optical density (OD) of the tested isolates before and after treatment with free EGCG and EGCG PLGA NCs (F2) at 620 nm using UV–vis spectrophotometer (SHIMADZU, Kyoto, Japan) at time intervals of 0, 1, 3, 5, 7, etc., to 24 h. Then, we constructed the bacterial growth curves via plotting log OD_620_ versus time (h).

### *In vivo* activities

#### Experimental protocol

Forty adult male albino mice (weight 21–25 g) were acquired from the animal house at Cairo University's College of Veterinary Medicine (Cairo, Egypt). The mice were acclimatized for one week before the experiment and kept under constant temperature and relative humidity control with 12-h light–dark cycles. Mice were fed ad libitum with regular food and water. The Ethical Committee for Laboratory Animals of Tanta University's Faculty of Pharmacy authorized all experimental techniques following the Guidelines for the Management and Use of Laboratory Animals (approval code TP/9/21-PR-003).

The mice were divided into five groups, each with eight mice, and treated for 10 days. The first group was used as a standard control group. A single injection of cisplatin (25 mg/kg i. p.) was given to the second group (Mi et al., 2018). The third group was treated orally with 100 mg/kg free EGCG once a day. The fourth and fifth groups were treated with placebo PLGA NCs or EGCG PLGA NCs (F2) once daily at an equivalent dose to free EGCG for 10 days. All third, fourth, and fifth groups receive the different treatment for 10 days (seven days before and three days after cisplatin injection) (Sahin et al., 2010; Pan et al., 2015).

#### Sample collection

Diethyl ether was used to anesthetize mice 72 h after cisplatin treatment. Blood samples were drawn and promptly centrifuged (4 °C, 300×*g* for 10 min) to separate the blood serum samples, which were then refrigerated at 20 °C for future analysis. One of the kidneys was immersed in 10% neutral buffered formalin for a histological investigation right away. The other kidney was instantly separated and frozen in liquid nitrogen for biochemical marker detection and RT-PCR analysis. According to the formula: relative kidney weight (%)=(kidney weight/body weight)×100%, relative kidney weight (%) was determined and statistically analyzed (Mi et al., 2018).

#### Determination of serum indices of nephrotoxicity

##### Serum creatinine

The assay relies on the Jaffe reaction, where a yellow/orange color forms when the metabolite is treated with alkaline picrate. The rate of the color development was proportional to the amount of creatinine in the sample, and the absorbance was measured at 490–500 nm according to the manufacturer protocol (Colorimetric Assay Kit 700460, Cayman, Ann Arbor, MI).

##### NGAL and kidney injury molecule-1 (KIM-1)

Lipocalin-2 (NGAL) Mouse ELISA Kit (ab119601, Abcam, Cambridge, UK) and – Mouse KIM-1 ELISA Kit (TIM1) ELISA^®^ Kit (ab213477, Abcam, Cambridge, UK) were used to estimate NGAL and KIM-1 respectively according to manufacturer protocol.

#### Assessment of oxidative stress markers

##### Lipid peroxidation

Lipid peroxidation levels were measured by assessing malondialdehyde (MDA) levels in the renal tissue homogenate using kits (Biodiagnostic, Giza, Egypt).

##### SOD activity

The superoxide dismutase enzyme activity in the renal homogenate was assessed following the manufacturer’s protocol, using a commercially available kit (Biodiagnostic, Giza, Egypt).

##### qRT-PCR for NLPR-3, NFκB, IL-1β, caspase-1, Bax, and Nrf-2 genes

According to the manufacturer's protocol, the manufacturer's protocol purified total RNA from kidney samples using TRIzols Reagent (Life Technologies, Carlsbad, CA). In a two-step RT-PCR experiment, 1 μg of total RNA was reverse-transcribed into single-stranded complementary DNA using the QuantiTects Reverse Transcription Kit (Qiagen, Germantown, MD) and a random primer hexamer. Maximas SYBR Green/Fluorescein qPCR Master Mix was used to amplify C-DNA amplicons through specific primers prepared according to manufacturer protocol (Supporting Table 1).

The melting curve analysis settings and thermal cycling were designed according to the previously reported study (Hazman et al., 2018). Finally, relative mRNA expression was measured using the 2^−ΔΔCT^ method and normalized to GAPDH (Livak & Schmittgen, 2001).

##### Histopathological examination of kidney sections

Following previously reported technique (Negm et al., 2022), kidney sections were prepared and stained then viewed under a light microscope.

### Statistical analysis

The data were provided as a mean ± standard deviation. Regression analysis was performed on all calibration curves, producing correlation coefficients. A one-way analysis of variance (ANOVA) was utilized to compare different groups, followed by a Tukey–Kramer’s post hoc test. *p*<.05 was used as the significant level. The statistical analysis was carried out using Prism version 6 (GraphPad Software, Inc., San Diego, CA).

## Results

### Structure elucidation of EGCG

**Figure 1.**
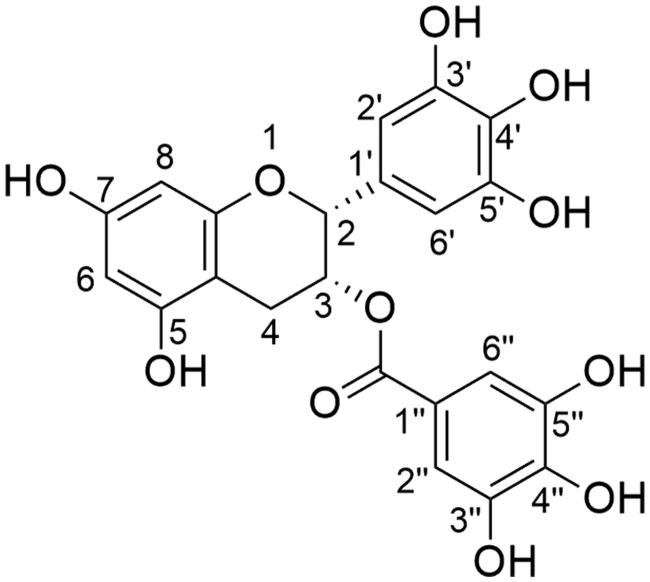
Chemical structure of EGCG.

The compound **I** was identified as (–)-epigallocatechin-3-*O*-gallate as chemical, ESI-MS, ^1^H, and ^13^C NMR data were compared to those described in the literature (Snitsarev et al., 2013; Selvi & Nagarajan, 2018). EGCG is a white amorphous powder with the chemical formula C_22_H_18_O_11_, established by ESI-MS 457.034 [M–H]^–^ and UV max = 275 nm. Figure 1 depicts the molecular structure of EGCG. ^1^H NMR (DMSO-d_6_, 500 MHz) and ^13^C NMR (DMSO-d_6_, 125 MHz) results are listed in Table 2.

**Table 2.** ^1^H NMR and ^13^C NMR (DMSO-d_6_, 500, and 125 MHz) for EGCG compound.

	Compound **I**	
	δ-H	δ-C
**2**	4.93 (s)	78.06
**3**	5.32 (s)	68.04
**4**	2.62 (d, *J*= 16 Hz)	25.74
	2.91 (dd, *J*= 16, 5 Hz)	
**5**		156.54
**6**	5.91 (d, *J*= 2.5 Hz)	95.47
**7**		155.62
**8**	5.81 (d, *J*= 2.5 Hz)	94.29
**9**		156.54
**10**		97.34
**1′**		132.37
**2′**	6.38 (s)	105.44
**3′**		145.44
**4′**		128.59
**5′**		145.44
**6′**	6.38 (s)	105.44
**1″**		117.95
**2″**	6.79 (s)	108.62
**3″**		146.66
**4″**		139.39
**5″**		146.66
**6″**	6.79 (s)	108.62
**Carbonyl**		165.25

### Aqueous core PLGA NCs

PLGA was employed as the main polymer in this study, and EGCG-PLGA NCs were formulated using a double emulsion/solvent evaporation technique. The sonication stage resulted in a w/o nanoemulsion consisting of an internal aqueous core surrounded with an organic polymer solution comprising Span 80. This primary nanoemulsion was mixed with Tween 80 and PVA in a second aqueous phase. The extra purpose of the PVA/water mixture was to increase the consistency of the external aqueous shell and aid the creation of nanodroplets allowing the production of stable NCs dispersion with mixed surfactants (Abdelkader et al., 2018).

**Figure 2.**
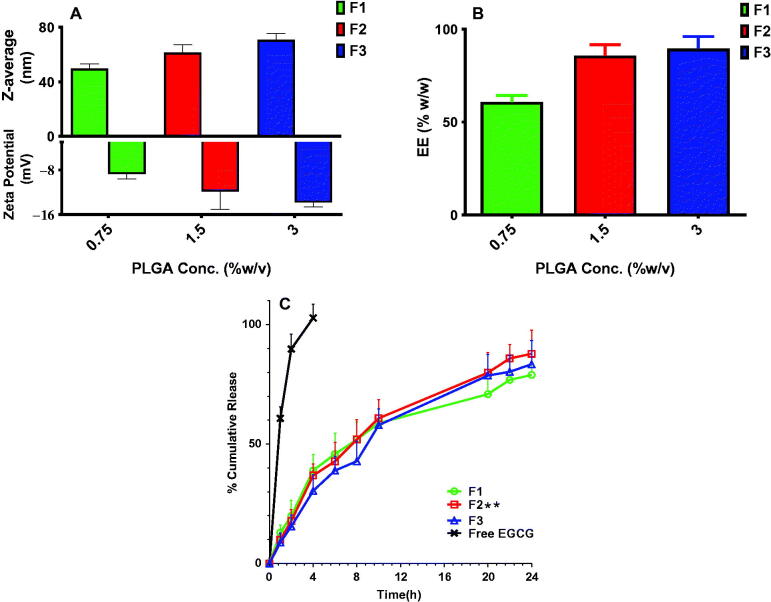
Physical and *in vitro* release characterization of EGCG PLGA NCs (F1–F3). Effect of PLGA concentration on (A) the particle size and zeta potential, (B) % entrapment efficiency, and (C) *in vitro* release pattern of free EGCG and EGCG PLGA NCs. Results show mean ± SD (*n* = 3) for A and B. For clarity, data are shown as mean ± SD (*n* = 3) for C. EGCG PLGA NCs (F2) have significantly higher initial burst and percent cumulative release (double asterisks, *p*<.05).

**Figure 3.**
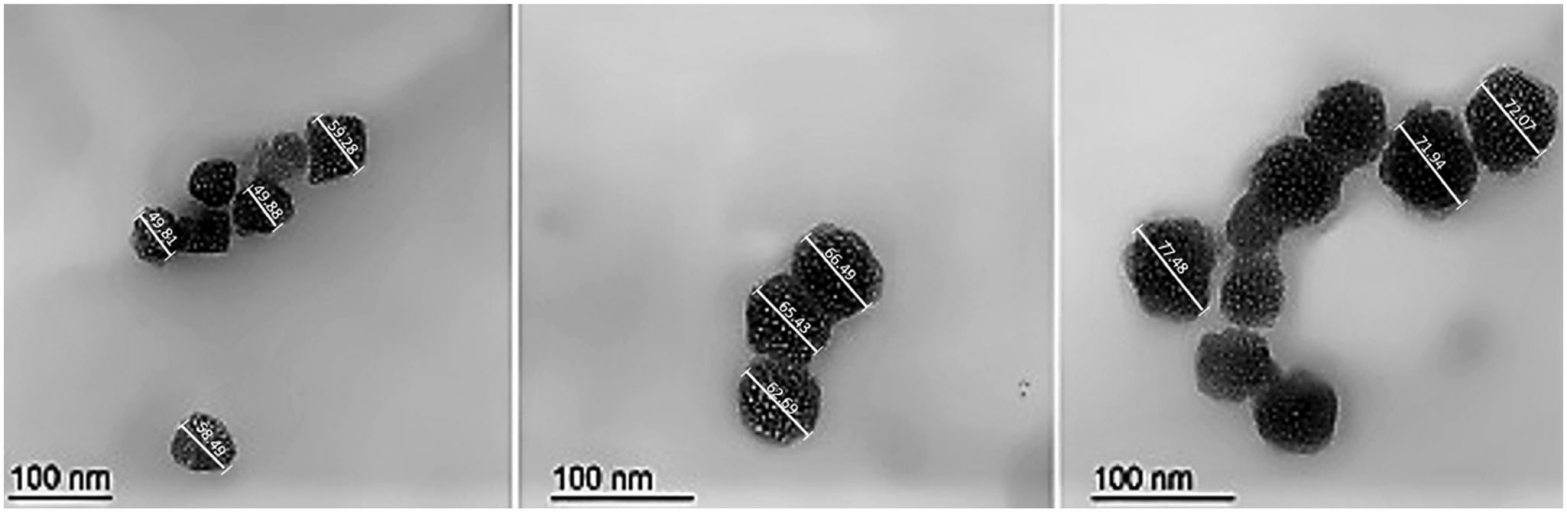
Transmission electron micrographs of EGCG PLGA NCs, respectively, A, B, and C for F1, F2, and F3. The measurements were displayed using Image J program (Bethesda, MD).

For instance, when Tween 80 and Span 80 were combined, stable and mono-dispersed NCs with a mean of particle size equal to 49.65 ± 3.31, 61.37 ± 5.90, and 70.76 ± 4.61 nm for F1, F2, and F3, respectively (Figures 2(A) and 3) have been obtained compared with significant bigger particle size produced in several previous studies (Almukainzi et al., 2022).

#### Effect of polymer concentration on physical properties of EGCG-PLGA NCs

Different PLGA concentrations of 0.75, 1.5, and 3%w/v were employed to prepare EGCG NCs (Table 1). Increasing PLGA concentration substantially (*p*<.05) produces NCs with larger particle sizes. The particle sizes of F1, F2, and F3 were 49.65 ± 3.31, 61.37 ± 5.90, and 70.76 ± 4.61 nm for PLGA concentrations of 0.75, 1.5, and 3%w/v, respectively, as shown in Figure 2(A). Additionally, a lower size distribution range could be attained at lower PLGA concentration and significantly (*p*<.05) increases with greater PLGA concentration. PDI values for F1, F2, and F3 are 0.099 ± 0.015, 0.125 ± 0.027, and 0.187 ± 0.03, respectively, for the same PLGA concentrations mentioned above.

The zeta potential of EGCG-PLGA NCs is heavily influenced by the concentration of PLGA (Figure 2(A)). Higher PLGA concentration increased the net negative surface charge of the resultant NCs (*p*<.05). F1, F2, and F3 have zeta potential values of −8.75 ± 0.88, −11.83 ± 3.22, and −13.82 ± 0.78 mV, respectively (Figure 2(A)). Figure 2(B) demonstrates that when PLGA concentration is increased from 0.75 to 3.0%w/v, EE has significantly (*p*<.05) raised from 60.85 ± 3.48 to 89.53 ± 6.51%w/w.

#### Morphological imaging

The geometrical structure of EGCG-PLGA NCs was visualized under TEM (Figure 3). The particle size of F1, F2, and F3 equaled 54.36, 64.87, and 73.83 nm (the particle size is calculated by computing the average of the individual particles displayed in Figure 3). PLGA NCs had a spherical nanostructure with a marked increase in particle size at higher PLGA concentrations. These results are very similar to the numerical values obtained by the zetasizer using the laser diffraction technical approach (Figure 2(A)).

#### In vitro release platform

EGCG-PLGA NCs had a biphasic release pattern, with an initial burst at four hours and a sustained release profile lasting 24 hours (Figure 2(C)). The percent cumulative release of free EGCG reached around 85% after two hours, indicating that the dialysis membrane had no long-term influence on *in vitro* release studies. When compared to free EGCG, all NC formulations had significantly (*p*<.05) more sustained release behavior (Figure 2(C)). The early burst of EGCE NCs with smaller diameters is considerably higher; for example, the initial bursts of F1 (49.65 ± 3.31 nm), F2 (61.37 ± 5.90 nm), and F3 (70.76 ± 4.61 nm) are 38.89 ± 6.72, 36.85 ± 4.79, and 30.41 ± 6.78, respectively (Figure 2(C)).

Furthermore, EGCG-PLGA NCs with higher percent EE had a more prolonged impact with greater percent cumulative release. F1 (EE = 60.85 ± 3.48%w/w) and F2 (EE = 85.79 ± 5.89%w/w) have total percent cumulative release of 78.89 ± 7.75 and 87.79 ± 9.84, respectively, as illustrated in Figure 2(C). F2 was chosen as the best formula for further *in vitro/in vivo* testing because it had the highest percent cumulative release, a high percent EE, well-controlled particle size, and adequate PDI and zeta potential values (Figure 2(A,B)).

### *In vitro* antibacterial activity

#### Susceptibility to free EGCG, EGCG PLGA NCs, and placebo PLGA NCs

The Kirby-Bauer method examined the bacterial susceptibility to free EGCG, EGCG PLGA NCs (F2), and placebo PLGA NCs. Free EGCG and EGCG PLGA NCs (F2) revealed antibacterial effects against the tested isolates. Meanwhile, placebo PLGA NCs did not exhibit any antibacterial activity. Thus, we determined the values of MICs, using the broth microdilution method, for free EGCG and EGCG PLGA NCs (F2) only. The values of MICs versus the tested isolates are shown in Table 3.

**Table 3.** Values of MICs of the free EGCG and EGCG-PLGA NCs against the tested isolates.

Bacterial isolates	*E. coli*	*K. pneumoniae*	*P. aeruginosa*	*P. mirabilis*
MIC value (µg/mL) of free EGCG	512	128	128	256
MIC value (µg/mL) of EGCG PLGA NCs (F2)	64	16	8	16

#### Growth curve

**Figure 4.**
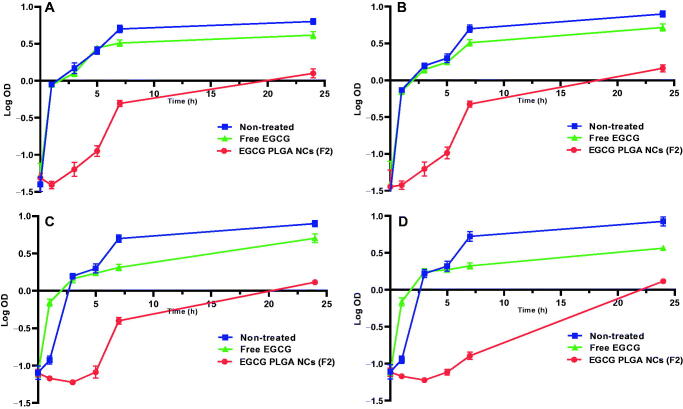
Growth curves of the tested isolates before and after treatment (A) *E. coli*, (B) *K. pneumoniae*, (C) *P. aeruginosa*, and (D) *P. mirabilis*.

We constructed growth curves for the tested isolates before and after treatment with free EGCG and EGCG PLGA NCs (F2) to study their effect on the growth process. Figure 4 shows that after treatment with EGCG PLGA NCs (F2), the isolates' growth was dramatically retarded (*p*<.05) compared to both the isolates treated with free EGCG and the non-treated isolates.

### *In vivo* activity

#### Kidney index

Cisplatin induced a marked increase in kidney index (48.1%) compared to the control group and this increase is significantly decreased by free EGCG and EGCG PLGA NCs (F2) treatment 22.8 and 31.92% respectively compared to cisplatin group. There was no significant difference between free EGCG and EGCG PLGA NCs treated groups (Table 4).

**Table 4.** Effects of EGCG pretreatment on kidney index in cisplatin-induced nephrotoxicity in mice.

	Bodyweight (g)		Kidney weight (g)	Kidney index
	Initial	Final		
Control	21.06 ± 0.43	27.16 ± 0.92	0.366 ± 0.016	1.347 ± 0.077
Cisplatin	23.26 ± 0.51	21.53 ± 0.73	0.420 ± 0.050	1.995 ± 0.220^a^
Free EGCG	21.84 ± 0.59	21.23 ± 0.82	0.330 ± 0.033	1.540 ± 0.098^b^
Placebo PLGA NCs	21.86 ± 0.41	19.89 ± 0.83	0.366 ± 0.044	1.850 ± 0.112
EGCG PLGA NCs (F2)	21.80 ± 0.49	22.22 ± 0.49	0.300 ± 0.014	1.358 ± 0.052^b^

Nephrotoxicity was induced by a single I.P. injection of cisplatin (25 mg/kg). Free EGCG or placebo PLGA NCs or EGCG PLGA NCs were given orally for 10 days.

Data expressed as mean ± SD (*n* = 8/group), significant difference vs. ^a^respective control, and ^b^respective cisplatin group each at *p* ˂ .05.

#### Serum indices of nephrotoxicity

Serum KIM-1 and NAGL levels are sensitive and reliable biomarkers of cisplatin-induced nephrotoxicity. Serum KIM-1 levels were substantially raised in the cisplatin group (233.33%) relative to the normal control group. Such rise in serum KIM-1 levels was significantly reduced in mice treated with free and nanoformulation of EGCG for 10 days (46.48 and 63.78%, respectively) compared to the cisplatin group.

**Figure 5.**
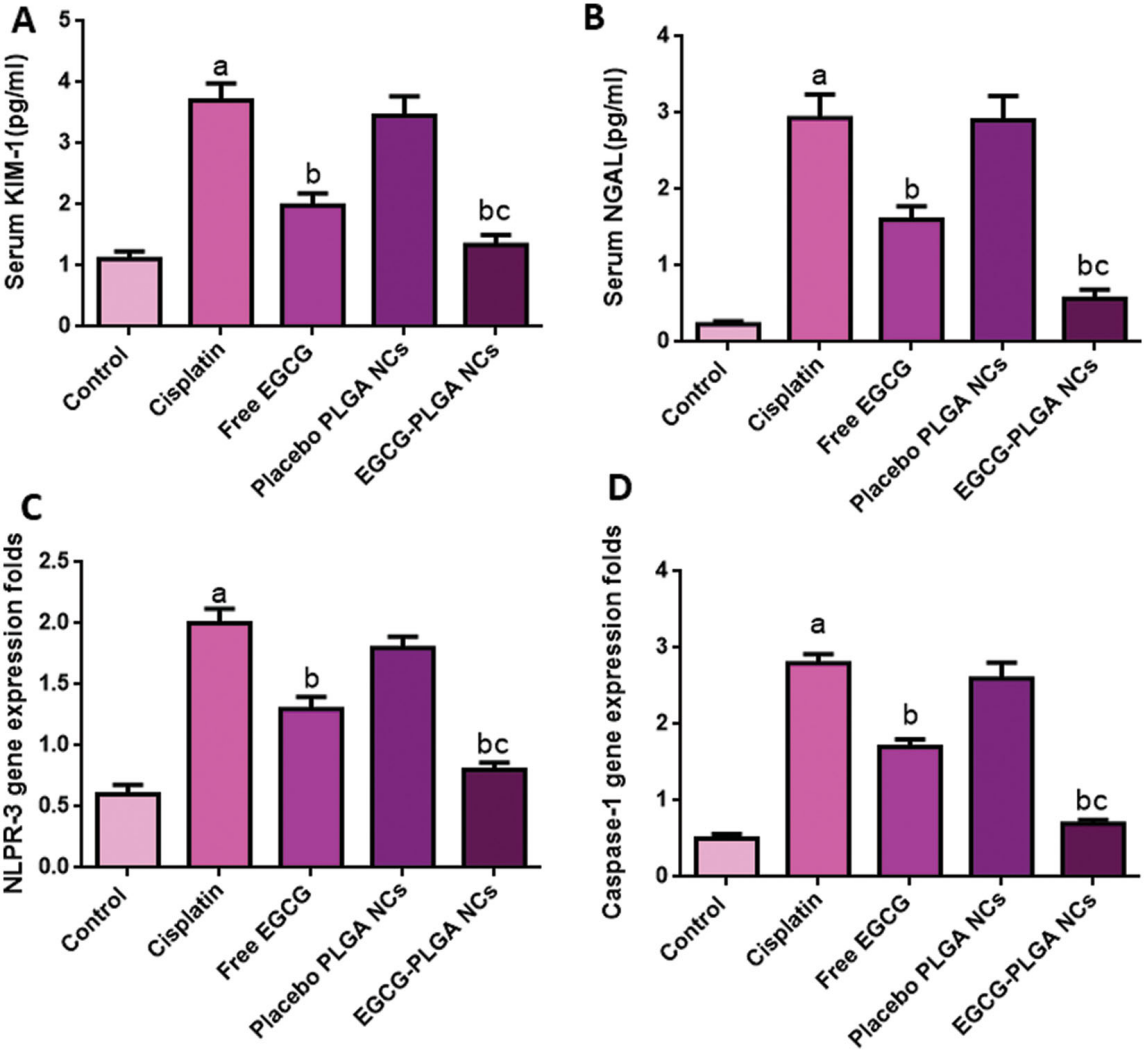
Effect of EGCG (free and encapsulated) pretreatment on (A) serum KIM-1 level, (B) serum NGAL level, (C) NLPR-3 gene expression level, and (D) caspase-1 gene expression level. Data expressed as mean ± SD (*n* = 8/group), significant difference vs. ^a^respective control, ^b^respective cisplatin group, and ^c^respective free EGCG group each at *p* ˂ .05.

Also, cisplatin-induced a marked increase in serum NGAL levels (1173.9%) compared to the control group, and this increase is significantly brought down by free EGCG treatment (45.39%). The effect was more pronounced in EGCG PLGA NCs (F2) group (81.54%) compared to the free EGCG group (Figure 5(A,B)).

As shown in Table 5, cisplatin treatment increased the serum levels of S. Cr (164.66%) compared to the control. Free EGCG pretreatment induced a remarked decrease in S. Cr level (37.21%). Also, EGCG PLGA NCs treatment induced greater reduction in S. Cr level (53.12%) compared to the cisplatin group (Table 5).

**Table 5.** Effects of EGCG Pretreatment on S. Cr level, kidney MDA level, and kidney SOD activity in cisplatin-induced nephrotoxicity in mice.

	S. Cr level (mg/dL)	Kidney MDA content (nm/g tissue)	Kidney SOD activity (U/mg tissue)
Control	1.33 ± 0.19	144 ± 14.50	2.75 ± 0.16
Cisplatin	3.52 ± 0.35^a^	248 ± 14.35^a^	1.25 ± 0.13^a^
Free EGCG	2.21 ± 0.24^b^	168 ± 9.14^b^	2.15 ± 0.28^b^
Placebo PLGA NCs	3.43 ± 0.32	245 ± 16.8	1.21 ± 0.14
EGCG PLGA NCs (F2)	1.65 ± 0.19^bc^	150 ± 7.31^bc^	2.6 ± 0.38^bc^

Nephrotoxicity was induced by a single I.P. injection of cisplatin (25 mg/kg). Free EGCG or placebo PLGA NCs or EGCG PLGA NCs were given orally for 10 days.

Data expressed as mean ± SD (*n* = 8/group), significant difference vs. ^a^respective control, ^b^respective cisplatin group, and ^c^respective free EGCG group each at *p* ˂ .05.

#### Renal oxidative stress markers

There was a pronounced increase in oxidative stress in the cisplatin group, as presented in Table 5. Compared to the normal control group, cisplatin-induced marked elevation in the renal lipid peroxidation manifested by a substantial increase in MDA content (72.22%). In addition, cisplatin showed pronounced suppression in renal SOD activity (54.54%). Pretreatment with free EGCG and EGCG PLGA NCs (F2) significantly alleviated oxidative stress markers and improved renal antioxidant enzyme capacity (72 and 108%, respectively). It significantly decreased MDA levels (32.25 and 39.51%, respectively) compared to the cisplatin group. EGCG PLGA NCs (F2) could almost completely abrogate MDA elevation and restored SOD activity to control levels (Table 5).

#### NLPR-3 inflammasome pathway

As displayed in Figure 5(C), cisplatin substantially upregulated NLPR-3 expression (233.33%) compared to normal control. Meanwhile, pretreatment with free EGCG and EGCG PLGA NCs (F2) enormously depressed the protein expression (35% and 60%) relative to the cisplatin group. The effect was more prominent in the EGCG PLGA NCs (F2) group.

**Figure 6.**
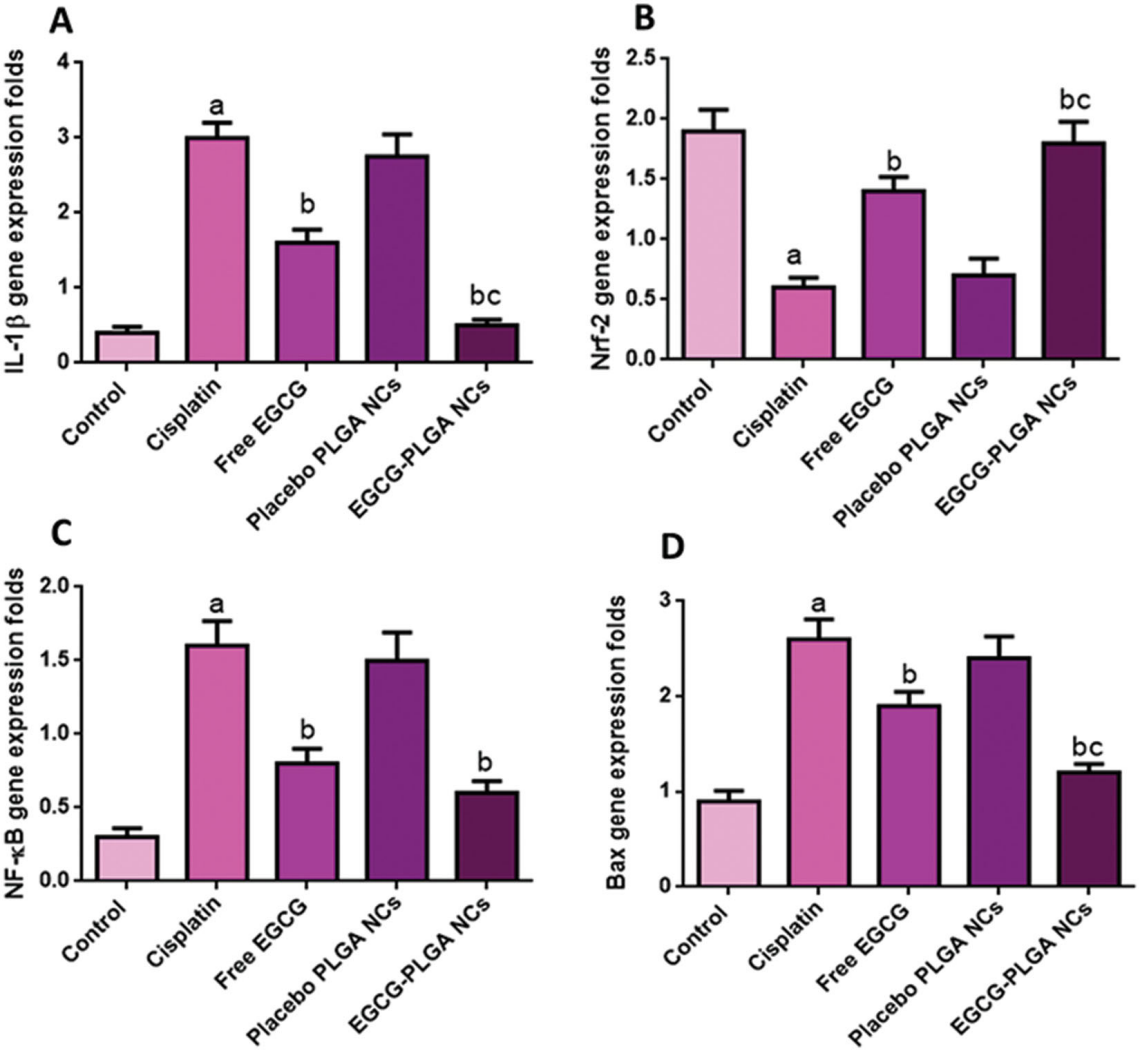
Effect of the EGCG (free and encapsulated) pretreatment on (A) IL-1β gene expression level, (B) Nrf-2 gene expression level, (C) NFκB gene expression level, and (D) Bax gene expression level. Data expressed as mean ± SD (*n* = 8/group), significant difference vs. ^a^respective control, ^b^respective cisplatin group, and ^c^respective free EGCG group each at *p* ˂ .05.

Compared to the control group, cisplatin-treated mice showed substantially higher caspase-1 expression levels (460%). Pretreatment with free EGCG and EGCG PLGA NCs (F2) produced a remarkable decrease in caspase-1 expression levels (39.28 and 75%) compared to the cisplatin group with a more remarked reduction in EGCG PLGA NCs (F2) group (Figure 5(D)) (*p*<.05). Figure 6(A) investigates that the cisplatin group had a substantial increase (650%) in IL-1β expression levels compared to the control group. Pretreatment with free EGCG and EGCG PLGA NCs significantly suppressed IL-1β expression levels (46.66 and 83.33%, respectively) relative to the cisplatin group with a more substantial effect in EGCG PLGA NCs group (Figure 6(A)) (*p*<.05).

#### Renal Nrf2 gene expression

In the current study, cisplatin significantly down-regulated Nrf2 expression in the renal tissue (68.42%) compared to the control group. This reduction in Nrf2 expression is an indicator of the depression of the antioxidant status of the kidney. Pretreatment with free EGCG and EGCG PLGA NCs (F2) upregulated Nrf2 mRNA expression (133.33 and 200%) compared to the cisplatin group (Figure 6(B)) with more pronounced effects in EGCG PLGA NCs (F2) group.

#### Renal expression of NFκB

The cisplatin group produced a remarkable up-regulation in renal NFκB gene expressions (433.3%) relative to the control group. Pretreatment with free EGCG and EGCG PLGA NCs treated groups showed a prominent downregulation in NFκB expression (50 and 62.5%) as a comparison with the cisplatin group. There was no significant difference between free EGCG and EGCG PLGA NCs treated groups (Figure 6(C)) (*p*<.05).

#### Renal expression of Bax gene

The cisplatin group produced a striking up-regulation in renal Bax gene expressions (188.88%) relative to the control group. Free EGCG pretreatment and EGCG PLGA NCs treated groups showed a prominent down-regulation in Bax expression (26.92 and 53.84%) compared to the cisplatin group with a more substantial effect in the EGCG PLGA NCs (F2) group (Figure 6(D)), (*p*<.05).

#### Kidney sections histopathological examination

**Figure 7.**
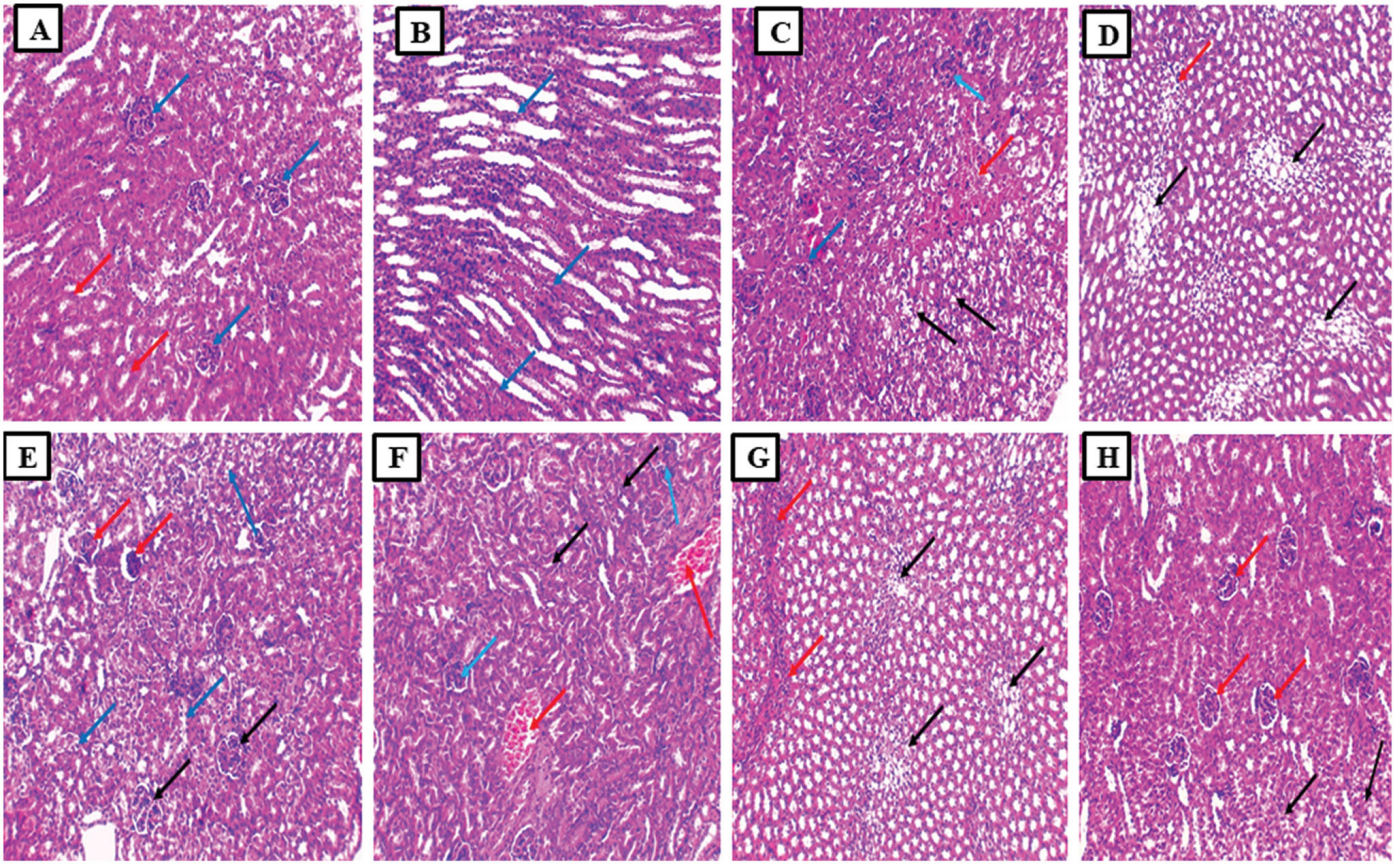
Histopathological examination of the kidney sections. (A) The section in the cortex of kidney of the normal control group showed average-sized glomeruli (blue arrows) surrounded by average-sized tubules lined with columnar cells (red arrows) (H and E, ×100). (B) Section in the medulla of kidney of the normal control group showed average-sized tubules lined with columnar cells (blue arrows) (H and E, ×100). (C) Section in the cortex of kidney of cisplatin group showed a focal area of necrosis (red arrow) surrounded by atrophic glomeruli (blue arrows) and tubules showing hydropic degeneration (black arrow) (H and E, ×100). (D) Section in the medulla of kidney of cisplatin group showed tubules with ballooning degeneration (marked hydropic degeneration) (black arrows) and focal inflammatory cellular infiltrate (red arrow) (H and E, ×100). (E) Section in the cortex of kidney of placebo PLGA NCs group showed congested vessels (red arrows) surrounded by atrophic tubules (atrophic tubules) and some atrophic glomeruli (blue arrows) and tubules showing hydropic degeneration (black arrow) (H and E, ×100). (F) Section in the medulla of kidney of placebo PLGA NCs group showed tubules with ballooning degeneration (marked hydropic degeneration) (black arrows) and inflammatory cellular infiltrate (red arrows) (H and E, ×100). (G) Section in the kidney of free EGCG group showed average-sized glomeruli (black arrows) and some atrophic glomeruli (red arrows) surrounded by average-sized tubules and some showing hydropic degeneration (blue arrows) (H and E, ×100). (H) Section in the kidney of EGCG PLGA NCs (F2) group showed average-sized glomeruli (red arrows) surrounded by average-sized tubules and few showing hydropic degeneration (black arrows) (H and E, ×100).

Examination of the kidney sections from the normal group displayed average-sized glomeruli surrounded by average-sized tubules lined with columnar cells (Figure 7(A,B)). While section in the cortex of the kidney of the cisplatin group showed a focal area of necrosis surrounded by atrophic glomeruli and tubules showing hydropic degeneration (Figure 7(C)) and showed tubules with ballooning degeneration (marked hydropic degeneration) and focal inflammatory cellular infiltrate (Figure 7(D)). Also, a section in the cortex of the kidney of the Placebo PLGA NCs group showed congested vessels surrounded by atrophic tubules (atrophic tubules) and some atrophic glomeruli and tubules showing hydropic degeneration (Figure 7(E)) and showed tubules with ballooning degeneration (marked hydropic degeneration) and inflammatory cellular infiltrate (Figure 7(F)). In addition, the section in the kidney of the free EGCG group showed average-sized glomeruli and some atrophic glomeruli surrounded by average-sized tubules and some showing hydropic degeneration (Figure 7(G)). Finally, a section in the kidney of the EGCG PLGA NCs (F2) group showed average-sized glomeruli surrounded by average-sized tubules and a few showing hydropic degenerations (Figure 7(H)).

## Discussion

There are many plant extracts and natural products used worldwide in traditional medicine to prevent and treat UTIs. Natural products have many advantages when used as therapeutic alternatives to antibiotics owing to their high safety, efficacy, and affordability (Elekhnawy et al., 2021; Tache et al., 2022). Recently, different nanotechnological strategies could be employed to increase the bioavailability of herbal medicines, hence enhancing their biological activities (Aslam et al., 2019). The existence of an aqueous core in PLGA polymeric colloidal carrier is the most significant innovation in our study since it could allow the effective trapping of highly aqueous soluble agents (Cosco et al., 2015). Our work described a new approach for the preparation of EGCG PLGA NCs that may entrap a high percentage of hydrophilic agents in the aqueous core of the NCs, providing a regular and sustained release manner. The formulation strategy commonly utilized to formulate aqueous core NCs is the double emulsion (w/o/w) – solvent evaporation technique, which leads to forming an interfacial polymeric layer deposited between an internal and external aqueous medium. Incorporating Tween 80 and Span 80 in the same nanoarchitecture resulted in the formation of NCs with well-controlled physicochemical features (Pavoni et al., 2020). Pharmaceutical tactics, including the incorporation of dual surfactants with varying HLB values, could effectively enhance the synthesis and stabilization of micro/nanoemulsions. The application of both hydrophilic and lipophilic surfactants can result in a surfactant additive effect. As a result, using a mixed-emulsified system can help small droplets form more efficiently (Pavoni et al., 2020). Mazarei et al. examined the impact of various Tween 80 (hydrophilic surfactant) and Span 80 (lipophilic surfactant) combinations on average particle diameter and physical stability (Mazarei & Rafati, 2019). Finally, they observed that a blended mixture with an HLB value equal to 10 had a significant stabilizing action against globules expansion caused by the Ostwald ripening. This suggests that the HLB value of a surfactant combination could be a key element in expecting the possibility of instability problems like the Ostwald ripening (Mazarei & Rafati, 2019).

Importantly, PLGA is the most significant formulation variable controlling the physical attributes of EGCG NCs, including particle diameter, zeta potential, and %EE. At higher PLGA concentration, greater viscosity of the organic interface acts as a barrier between the internal and external aqueous phases restricting the diffusion of EGCG out to the surrounding aqueous phase (Abdelkader et al., 2018), leading to EGCG PLGA NCs with much higher percent EE (Figure 2(B)). Furthermore, the amount of EGCG enclosed in prepared NCs is influenced by the PLGA content. Increasing PLGA concentration could produce heavier consistency of the primary emulsion’s background, which requires higher shear force and faster homogenization speed to break down more stiff oily globules. The processing parameters adjusted throughout the study were kept constant, resulting in a coarser emulsion with larger oily droplets/globules homogenously dispersed in the second emulsion at higher PLGA concentration, leading to a bigger particles size (Figure 2(A)). Furthermore, increasing PLGA concentration induces more carboxylic acid groups in EGCG NCs dispersion, resulting in a substantial increase in negative zeta potential, which enhances stronger inter-particles repulsion and stabilization (Figure 2(A)) (Abdelkader et al., 2018; Almukainzi et al., 2022).

The study of drug release through the dialysis membrane is a well-documented experiment that ensures the complete separation of drug molecules coated with polymeric matrix in the donor from the receiver side (Almukainzi et al., 2022). Free EGCG release had a quick and complete release due to its extreme hydrophilicity (Granja et al., 2019) (Figure 2(C)), indicating that the dialysis membrane technique does not provide extra sustained action. SLS was added to the release medium to improve EGCG passage out of the polymeric network maintaining the sink condition required for continuous release over 24 hours (Almukainzi et al., 2022). The majority of previous studies suggest that nanovesicular existence loaded with active drugs is transported directly into the bloodstream via Peyer's patches pathway (da Silva et al., 2020). pH = 7.4 was used in our *in vitro* release investigation to mimic *in vivo* circumstances and investigate the release behavior of EGCG in a pH similar to blood circulation.

For all PLGA NCs (F1–F3), the release of EGCG has occurred over two stages (biphasic release behavior) (Figure 2(C)). The initial burst occurred after four hours and shot around 30–39% of the EGCG deposited on the external coat of NCs into the releasing medium. The second phase, which has a long-term release, is due mostly to the gradual migration of EGCG out of the PLGA network and/or progressive erosion that may occur through the polymeric matrix, allowing EGCG to be separated more easily (Abdelkader et al., 2018). The influence of PLGA concentration on release patterns could be attributable to its significant effect on particle size and %EE. EGCG PLGA with smaller particle sizes has a more intense initial burst, whereas NCs encasing a larger amount of EGCG have a higher percent cumulative release and a longer-lasting effect (Almukainzi et al., 2022).

We investigated the antimicrobial activity of the free EGCG and EGCG PLGA NCs (F2). Placebo PLGA-NCs did not exhibit any antimicrobial activity; meanwhile, the free EGCG and EGCG PLGA NCs (F2) showed MIC values against the tested isolates that ranged from 128 to 512 µg/mL and from 8 to 64 µg/mL, respectively. Furthermore, EGCG PLGA NCs (F2) resulted in remarked retardation in bacterial growth (*p*<.05). A finding means that EGCG-PLGA NCs affected various physiologic processes in the bacterial cells. The growth curve of bacteria is commonly utilized to evaluate the influence of the antibacterial agents over 24 h. These findings prove that the antibacterial activity of EGCG-PLGA NCs is higher than free EGCG against the tested uropathogenic bacteria.

Nephrotoxicity is a major medical problem associated with cisplatin administration (Pan et al., 2015); this study aims to determine whether EGCG PLGA NCs can lessen cisplatin-induced renal side effects and assess their effectiveness in tissues protection against injury. Renal function loss is clearly observed because of nephrotoxicity, as seen by increases in serum creatinine, blood urea nitrogen (BUN), and neutrophil gelatinase-associated lipocalin (NGAL) (Luo et al., 2021). In cisplatin-treated mice, there was a significant decrease in body weight and an increase in kidney index, serum creatinine, and NGAL, which corresponded to the cisplatin-induced loss of renal function. In addition, nephrotoxic biomarkers such as KIM-1 have been employed to indicate renal dysfunction (Lee et al., 2019).

In the current study, cisplatin induced significant nephrotoxicity manifested by a pronounced increase in S. Cr, NGAL, and KIM-1 serum levels and a marked increase in kidney index. EGCG pretreatment provided significant renoprotection and significantly improved renal toxicity markers, and these results are consistent with previous results (Pan et al., 2015). In addition, the effects were more promising with nanoformulation of EGCG than free EGCG, confirming the beneficial effects of nano-drug delivery.

Invading pathogens or injured tissues generate inflammation, a vital host response. A moderate inflammatory response aids the restoration of injured tissue. Prolonged inflammation, on the other hand, may increase tissue damage and inflammatory cytokine overproduction. IL-1β is a master inflammatory cytokine that plays a crucial role in cell activation and the production of other inflammatory cytokines and chemokines. It is produced by a multiprotein complex called the inflammasome. The NLRP3 inflammasome, which contains the NLRP3 sensor, ASC adaptor, and caspase-1 protease, is one of the most researched inflammasomes (Jiang et al., 2012). NLRP3 inflammasome components form massive cytoplasmic complexes in response to stimulation, and caspase-1 activation leads to IL-1β maturation and secretion. NLRP3 inflammasome activation may be followed by caspase-1-mediated fast cell death and cytokine production (Joo et al., 2012). The role of the NLPR-3 signaling axis in nephrotoxicity has not been intensely studied before. Renal inflammation has been connected to the activation of the NLRP3 inflammasome and the overproduction of IL-1β in a unilateral ureteral obstruction mouse model. *In vivo* studies have shown that EGCG can suppress the development of lupus nephritis (Tsai et al., 2011).

In addition, EGCG was helpful in a rat model of contrast-induced nephropathy (CIN) (Gao et al., 2016). They also discovered that EGCG lowers IL-1β secretion and NLRP3 protein expression. The effect of the NLPR-3 inflammasome signaling axis in the pathophysiology of cisplatin-induced nephrotoxicity has not been explored yet. Thus, this study aims to investigate whether this pathway mediates the renoprotective effects of EGCG in cisplatin-induced nephrotoxicity.

In the current study, cisplatin-induced a significant upregulation of NLPR-3 expression and its downstream signaling axis IL-1β/caspase-1 and the treatment with EGCG PLGA NCs strongly suppressed NLPR-3 inflammasome pathway activation. These findings are consistent with earlier research (Tsai et al., 2011; Gao et al., 2016).

Several studies have stated the inhibitory effects of EGCG on the NLPR-3 axis (Tőzsér & Benkő, 2016; Lee et al., 2019; Castejón-Vega et al., 2020; Luo et al., 2021). Tsai et al. (2011) reported that the activity of the renal NLRP3 inflammasome was decreased by EGCG, as were the expressions of NLRP3, caspase-1, and IL-1β, perhaps due to the EGCG-induced attenuation of NFκB pathway activity.

The effects on the inflammasome pathway were more crucial with EGCG PLGA NCs formulation than free EGCG, confirming the beneficial effects of nano-drug delivery. Ellis et al. reported that EGCG inhibits NFκB activity and lowers IL-1β release. The downregulation of NLRP-3 inflammasomes and reduced caspase-1 activation is linked to lower levels of IL-1β (Ellis et al., 2011).

Cisplatin-induced nephrotoxicity is associated with marked inflammation manifested by a considerable increase in renal IL-1β and EGCG treatment significantly decreased kidney IL-1β levels, and these findings are in line with earlier research (Pan et al., 2015; Castejón-Vega et al., 2020; Yang et al., 2020).

Nrf2 is translocated into the nucleus and induces antioxidant genes expression and decreased tissue damage due to oxidative stress (Shi et al., 2018). This pathway can boost phase II enzymes' anti-oxidative activity, which can help to reduce inflammation (Ye et al., 2015).

In the current study, cisplatin-induced marked suppression of Nrf-2 expression and pretreatment with EGCG PLGA NCs markedly restored Nrf-2 expression levels and enhanced the antioxidant capacity of the kidney. These results follow previous studies (Ye et al., 2015; Kanlaya et al., 2016; Pan et al., 2017).

A previous study (Tsai et al., 2011) presented that the antioxidant pathway Nrf2 was activated by EGCG administration, which decreased renal oxidative stress. Further, the existing reports demonstrated that oxidative stress plays a notable role in NLRP3 inflammasome activation (Abais et al., 2015). Hence, inhibition of oxidative stress can abrogate NLPR3 inflammasome activation.

Our study showed EGCG treatment significantly induced Nrf2 expression and inhibited cisplatin-induced oxidative stress. However, our findings indicated that the inhibition impact of EGCG on NLRP3 inflammasome activation in the kidney could be exerted through modulating the Nrf2 antioxidant pathway.

In the current study, oxidative stress is confirmed by a significant increase in MDA levels and suppressing kidney SOD activity. Treatment with EGCG PLGA NCs (F2) strongly ameliorated cisplatin-induced oxidative stress, decreased MDA levels, and upregulated SOD activity, and these results are confirmed by several reports (Pan et al., 2015; Tőzsér & Benkő, 2016; Yang et al., 2020; Luo et al., 2021).

NFκB is released and translocated into the nucleus due to oxidative stress. Accordingly, it binds to DNA and increases the transcription of many inflammatory genes, including cytokine and chemokine genes (Cichoż-Lach & Michalak, 2014). In the current study, cisplatin treatment induced significant activation of the NFκB pathway upon cisplatin injection following the previous studies (Jiang et al., 2012; Joo et al., 2012; Siracusa et al., 2021). EGCG pretreatment had a strong inhibitory effect on the NFκB activation pathway. The nanoformulation group reduced NFκB expression nonsignificantly compared to free EGCG.

Bcl-2 family proteins regulate physiological and pathological apoptosis. The family includes cell death promoters like Bax and Bad and cell death inhibitors like Bcl-2, Bcl-X, etc. It has been shown that a high Bax/Bcl-2 ratio is linked to increased apoptotic activation vulnerability (Lee et al., 2008).

In the current investigation, cisplatin provoked a marked apoptotic death of the renal cells through upregulating Bax. Pretreatment with EGCG PLGA NCs strongly decreased Bax expression levels. EGCG appears to have an antiapoptotic effect in cisplatin-induced apoptosis by modulating the Bcl-2 family of proteins (Ann Beltz et al., 2006). These results are consistent with other research that confirmed the antiapoptotic effects of EGCG (Liu et al., 2017).

## Conclusions

EGCG-PLGA NCs enhanced the antibacterial activity of free EGCG against the tested uropathogens. This finding could be one of the solutions for the spreading of antibiotic resistance, especially among uropathogenic bacteria. Additionally, EGCG PLGA NCS exerted promising reno-protective effects in mice's cisplatin-induced nephrotoxicity, as evidenced by a significant decrease in kidney index, S. Cr, KIM-1, and NGAL serum levels, and improved histopathological changes associated with cisplatin nephrotoxicity. The underlying mechanisms of such protective effects may be mediated via modulation of the NFκB/NLPR-3 inflammasome pathway and amelioration of oxidative stress. These results confirm that aqueous core PLGA considered a well-functional nanocarrier system for efficient drug delivery could be employed for multiple clinical applications in the future.

## Supplementary Material

Supplemental Material
