# Supplementary material for: Aqueous core epigallocatechin gallate PLGA nanocapsules: characterization, antibacterial activity against uropathogens, and in vivo reno-protective effect in cisplatin induced nephrotoxicity
Source: Drug Deliv. 2022 Jun 16;29(1):1848–62. doi: 10.1080/10717544.2022.2083725 (PMC9225707; doi:10.1080/10717544.2022.2083725)
Supplement: Supplemental Material [file IDRD_A_2083725_SM3479.docx]

**Supplementary Table1.** Primers used and their sequence

| Target gene | Probe | References |
| --- | --- | --- |
| NLRP3 | 5ʹ-GCA CTG CTG AGG CTC TCT C-3ʹ  5ʹ-GTA GAA GTG CTC AGC CCC AG-3ʹ | Shi et al., 2021 |
| Caspase-1 | F-5′-GAA AAG GCA CGA GAC CTG TGC-3′  R-5′-CTT GAG GGA ACC ACT CGG TCC-3′ | Hazman et al., 2018  Meier et al., 2019 |
| IL-1β | ACT CCT TAG TCC TCG GCC A  TGG TTT CTT GTG ACC CTG AGC |  |
| Nrf2 | CCATGCCTTCTTCCACGAA  AGGGCCCATGGATTTCAGTT | Espinosa et al., 2014 |
| NFKB | F-5′TCCCCAAGCCAGCACCCCAGC′3  R-5′GGCCCCCAAGTCTTCATCAGC′3 | Hazman et al., 2018 |
| Bax | 5′-AGCTCT GAGCAGATCATGAAG-3′  5′-GGTGGACGC ATCCTGAG-3′ | Jing-Jing et al., 2012 |
| GADPH | 5′-AGAAGG CTGGGGCTCATTTG-3′  5′-AGGGGCCAT CCACAGTCTTC-3′ | Jing-Jing et al., 2012 |
